# The p130Cas-Crk/CrkL Axis: A Therapeutic Target for Invasive Cancers Unveiled by Collaboration Among p130Cas, Crk, and CrkL

**DOI:** 10.3390/ijms26094017

**Published:** 2025-04-24

**Authors:** Pegah Farhadi, Taeju Park

**Affiliations:** 1 Medical Biology Research Center, Health Technology Institute, Kermanshah University of Medical Sciences, Kermanshah 67155, Iran; 2 Children’s Mercy Research Institute, Children’s Mercy Kansas City, Kansas City, MO 64108, USA; 3 Department of Pediatrics, University of Missouri-Kansas City School of Medicine, Kansas City, MO 64108, USA

**Keywords:** p130Cas, Crk, CrkL, p130Cas-Crk/CrkL axis, cancer cells, normal cells, cell migration, motility, invasion

## Abstract

Numerous studies have documented the involvement of p130Cas (Crk-associated substrate) in a wide range of cellular processes across different types of cells. These processes encompass cell transformation, the connection between the extracellular matrix and the actin cytoskeleton, cell migration and invasion, and cardiovascular development. Moreover, p130Cas has been associated with the regulation of various physiological processes, including mammary, bone, brain, muscle, and liver homeostasis. The diverse functions of p130Cas can be attributed to its possession of multiple protein–protein interaction domains, which sets it apart as a unique class of adaptor protein. It is well established that p130Cas interacts critically with the CT10 regulator of kinase (Crk) adaptor protein family members, including CrkII, CrkI, and Crk-like (CrkL), which is the basis for the naming of the Cas family. The Crk family proteins play a crucial role in integrating signals from various sources, such as growth factors, extracellular matrix molecules, bacterial pathogens, and apoptotic cells. An increasing body of evidence suggests that the dysregulation of Crk family proteins is linked to various human diseases, including cancer and increased susceptibility to pathogen infections. This review focuses primarily on the structural and functional aspects of the interaction between p130Cas and the Crk family proteins, providing insights into how these proteins regulate specific signaling events. Furthermore, we delve into the functions of p130Cas and the Crk family proteins in both normal and tumor cells to gain a comprehensive understanding of their collaborative roles in cellular physiology and pathology. This review demonstrates that tumor cell migration and invasion are the two cellular functions that have been studied the most for the p130Cas-Crk/CrkL axis. Understanding the tumor cell migration and invasion that require both p130Cas and Crk/CrkL is necessary to further evaluate the role of the p130Cas-Crk/CrkL axis in cancer. Establishing the contribution of the p130Cas-Crk/CrkL axis to cancer will facilitate the development of cancer drugs targeting the axis to inhibit cancer cell dissemination and improve patient outcomes.

## 1. Introduction

The Crk-associated substrate (Cas) family of adaptor proteins has become prominent with extensively linked signaling nodes that play crucial regulatory roles in both normal and pathological cellular processes. These proteins are distinguished by the existence of numerous conserved motifs that facilitate protein–protein interactions and by significant tyrosine and serine phosphorylation [1]. The Cas family comprises five distinct adaptor proteins: p130Cas/breast cancer anti-estrogen resistance protein 1 (BCAR1), neural precursor cell expressed, developmentally downregulated 9 (NEDD9), human enhancer of filamentation-1 (HEF-1 or Cas-L), embryonal Fyn-associated substrate (EFS), and Cas scaffolding protein family member 4 (CASS4). Similar modular structures with various interaction domains and multiple tyrosine and serine phosphorylation motifs are shared by the Cas family members [2]. The involvement of p130Cas in a multitude of cellular processes across different cell types has been documented. These processes include cell transformation [3], connection to the extracellular matrix (ECM) through the actin cytoskeleton [4], integrin signaling [5], growth factor–receptor signaling [6], antigen–receptor signaling [7], cell migration and invasion [8], bacterial invasion [9], and cardiovascular development [3]. The diverse functions of p130Cas are attributed to its possession of multiple protein–protein interaction domains [10]. Furthermore, p130Cas has been linked to the regulation of various physiological processes, such as mammary, bone, brain, muscle, and liver homeostasis [11].

p130Cas, the most widely expressed member of the Cas family, was identified as a 130 kDa protein that undergoes significant tyrosine phosphorylation in cells expressing p47 v-Crk (CT10 regulator of kinase) and p60 v-Src (sarcoma) oncoproteins [12,13]. It was observed that tyrosine-phosphorylated p130Cas was localized in both membrane and nuclear fractions upon subcellular fractionation. On the other hand, the majority of unphosphorylated p130Cas was found in the cytoplasm [12]. While lacking a kinase domain, p130Cas possesses several protein–protein interaction domains that facilitate its association with various binding partners [1]. Acting as an adaptor protein in multiprotein complexes, p130Cas plays a crucial role in integrating signals from the ECM environment, soluble ligands, and mechanical stress [2]. A variety of proteins that are involved in cell movement, such as Crk, focal adhesion kinase (FAK), protein tyrosine kinase 2 (PYK2), non-catalytic region of tyrosine kinase (Nck), and Src, interact with p130Cas, suggesting that p130Cas plays a central role as a signaling center in regulating cell motility [1]. Further, the phosphorylation of p130Cas plays a significant biological role, which is further intriguing due to the association of p130Cas with a wide range of ECM molecules, growth factors, hormones, and metabolic intermediates [1]. These molecules induce tyrosine phosphorylation of p130Cas and subsequently facilitate its coupling to the Crk adaptor protein family. The naming of the Cas family is based on the interaction with members of the Crk adaptor protein family [14]. The substrate domain of p130Cas contains multiple YxxP motifs, which mediate the binding to the Crk family adaptor proteins. This protein–protein interaction enables the diversification and amplification of signaling inputs [1].

The Crk family adaptor proteins are cellular counterparts of v-Crk and consist of Crk and Crk-like (CrkL). These members are composed of Src homology 2 (SH2) and Src homology 3 (SH3) domains, which play a crucial role in the assembly of macromolecular protein complexes and facilitate their localization to specific regions within the cell [15]. Crk family proteins play a critical role in integrating signals from diverse sources, such as growth factors, ECM molecules, bacterial pathogens, and apoptotic cells. A growing body of evidence suggests that the dysregulation of Crk and CrkL is linked to various human diseases, such as cancer and pathogen infections [13]. Crk and CrkL participate in cellular processes, such as cell growth, migration, and adhesion, by engaging in interactions with numerous proteins through their SH2 and SH3 domains. These interactions are frequently initiated by the activation of receptor or non-receptor tyrosine kinases in signal transduction pathways. This mechanism ensures that cells react appropriately to a wide range of external stimuli [16].

In terms of the signal transduction pathway, Crk family proteins serve as indispensable components that enhance and reinforce FAK/Src signaling through the small GTPase Ras-related protein-1 (Rap1). This process results in the continuous activation of FAK/Src, leading to increased invasiveness, chemoresistance, epithelial–mesenchymal transition (EMT), and ultimately, unfavorable prognosis for patients [17]. Research conducted in recent years has provided evidence that the assembly of the molecular signaling scaffold p130Cas-Crk/CrkL plays a crucial role in the organization of the actin cytoskeleton, which in turn, leads to changes in various cellular processes, such as cell migration [18], invasion [19], phagocytosis, and survival [20,21]. This review primarily focuses on the structural and functional aspects of the interaction of p130Cas and Crk family proteins, shedding light on how these proteins regulate specific signaling events. Additionally, we delve into the shared functions of p130Cas and Crk family proteins in both normal cells and tumor cells to understand their common roles in cellular physiology and pathology.

## 2. Structures of p130Cas and Crk Family Proteins

**Figure 1:**
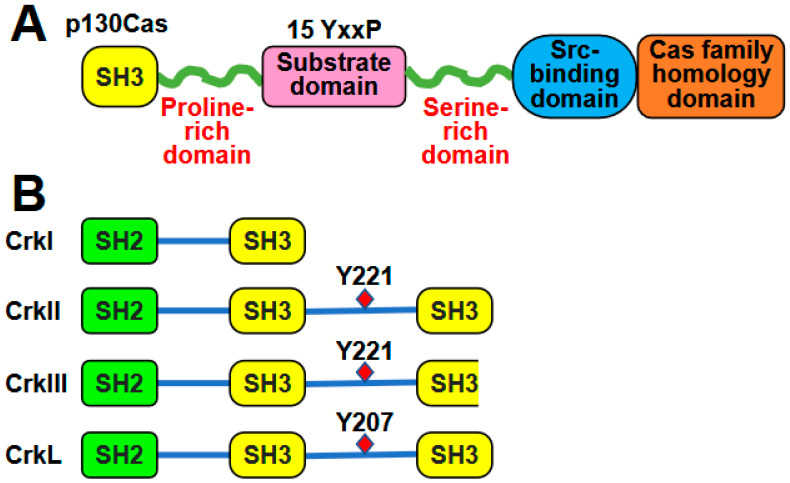
Structures of p130Cas (**A**) and Crk family proteins (**B**). [Drawn using Microsoft PowerPoint].

The role of p130Cas is closely associated with its structural organization, indicating its function as an adaptor protein. Structurally, the amino (N)-terminus of p130Cas comprises a SH3 domain, a proline-rich domain, and a substrate domain (Figure 1A). The substrate domain is made up of 15 repeats of the YxxP consensus phosphorylation motif for Src family kinases (SFKs) [13]. Proteins possessing SH2 domains bind to phosphotyrosine residues in this domain. The YxxP motifs are identified by the three amino acids succeeding the phosphotyrosine. The repetitive occurrence of YxxP motifs likely contributes to signal amplification and diversification. The presence of multiple YxxP motifs in p130Cas has sparked ongoing interest and speculation due to its unique arrangement. These motifs, when phosphorylated, create a consensus binding site for the SH2 domain of Crk family proteins. Intriguing research has demonstrated that the substrate domain of p130Cas is phosphorylated in a processive manner when Src binds to the polyproline region of p130Cas. This initial finding suggests that the sequential addition of phosphates may establish a pattern that prioritizes a signaling mechanism. The close proximity and repetition of the YxxP motifs in p130Cas primarily serve to amplify signals and maximize the output of the Crk-mediated pathways [22]. Additionally, this arrangement also contributes to signal diversification, as the functional cooperation within the Crk assemblages expands the range of output signals. This finding is particularly relevant during the integrin-mediated spreading and migration on ECM molecules [13]. Adjacent to the substrate domain is the serine-rich domain, which adopts a four-helix bundle conformation. This domain serves as a protein-interaction motif, similar to those observed in other adhesion-related proteins, such as FAK and vinculin [23]. The remaining carboxy-terminal sequence encompasses a bipartite Src-binding domain (residues 681–713) that has the ability to interact with both the SH2 and SH3 domains of Src [1] (Figure 1A).

The Crk family represents the original form of a unique group of regulatory proteins known as adaptors. These adaptors are made up of distinct SH2 and SH3 domains, which are connected by flexible linker sequences. These domains and linkers serve as fundamental components of multiprotein complex formation. As the term “adaptor” suggests, these molecules play a crucial role in physically connecting tyrosine phosphorylated proteins to different intracellular signaling pathways [13]. The Crk gene encodes for three different translation products through alternative splicing: CrkI, CrkII, and the later discovered CrkIII [24] (Figure 1B). CrkII is a 42-kDa protein that contains one SH2 domain and two SH3 domains, CrkSH3(N) and CrkSH3(C). The SH3 domains are separated by a spacer region harboring a negative regulatory tyrosine residue at 221 (Y221). CrkI shows 60% sequence similarity to CrkII but differs primarily by lacking the second SH3 domain. Additionally, CrkI does not possess the Y221 negative regulatory site and is associated with higher levels of tyrosine-phosphorylated proteins, compared to CrkII [25].

The differences in the transforming abilities of CrkII and CrkI are partly due to a specific post-translational modification involving phosphorylation on Y222 in chicken (Y221 in mouse and human) [26]. CrkL has a tyrosine residue at 207 (Y207) that is comparable to Y221. Both CrkII and CrkL are regulated in a negative manner through mechanisms involving autoinhibitory phosphorylation. This autoinhibitory phosphorylation ultimately hinders the ability of CrkII and CrkL to function as an adaptor protein. This process can be compared to the phosphorylation of the C-terminal tyrosine in Src family kinases by C-terminal Src kinase (CSK). This phosphorylation allows Src to adopt a “closed” conformation involving the Src SH2 domain and a C-terminal phosphorylation motif. Similarly, the phosphorylation of Y221 in Crk (or Y207 in CrkL) led to an interaction between the linker region and the SH2 domain. This intramolecular interaction sequestered the SH2 and SH3 domains, preventing them from binding to target proteins [27]. The discovery and characterization of CrkIII, which differs from CrkII mainly because of its truncated C-terminal SH3 domain, may potentially shed light on the elusive function of this specific domain. The Crm1-binding site located in the CrkSH3(C) is missing in CrkIII. Consequently, CrkIII is unable to bind to this nuclear export factor. This discovery suggests that CrkIII may be localized in the nucleus of human mesangial cells. It also raises questions regarding the biological role of nuclear-localized CrkIII [24]. However, the lack of follow-up studies of CrkIII makes its general biological significance unclear.

CrkL, which has a similar overall structure to CrkII, consists of one SH2 domain and two SH3 domains [24]. Sequence comparison reveals significant homology to CrkII. The SH2 domain of CrkL appears to exhibit the same specificity to YxxP motifs and interacts with similar sets of proteins as the CrkII SH2 domain in vitro [15]. This finding suggests that, in many instances, Crk and CrkL proteins may have similar functions [28]. In fact, overlapping roles of both proteins have been studied extensively in various biological processes (reviewed in [29]). According to the National Center for Biotechnology Information (NCBI) Protein BLAST, the amino acid comparison reveals that human CrkII and CrkL share 57% identity, while mouse CrkII and CrkL exhibit 56% identity [29]. Interestingly, the comparison between human and mouse CrkII and CrkL demonstrates remarkable conservation, with 99% and 97% identity, respectively. This finding suggests that these proteins are highly conserved between the two species, emphasizing their significance in biological processes.

## 3. p130Cas-Crk/CrkL Interaction

**Figure 2:**
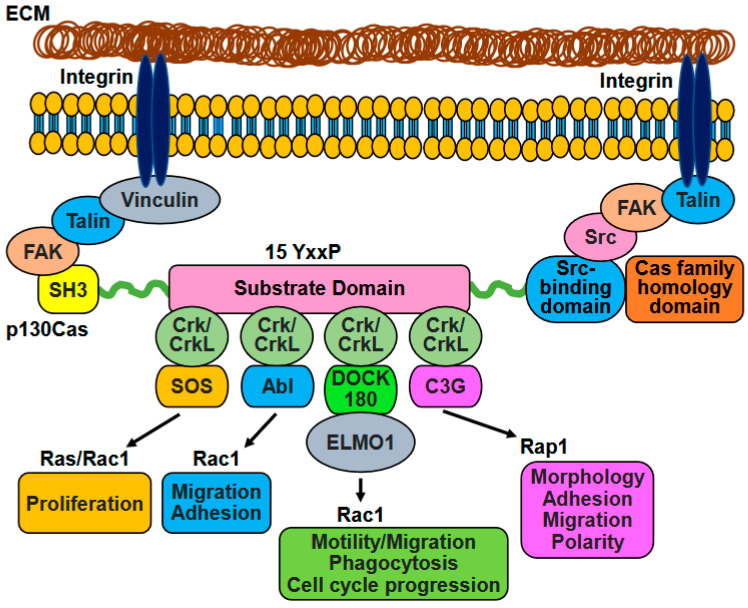
Interactions between p130Cas and Crk/CrkL lead to various complex formation and functions. [Drawn using Microsoft PowerPoint].

The interaction between Crk family proteins and p130Cas is influenced by the phosphorylation states of both proteins. This interaction involves the coordinated coupling and uncoupling of the p130Cas-Crk/CrkL complex (Figure 2). The SH2 domains of Crk and CrkL bind to phosphotyrosine residues found within the YxxP motifs of the substrate domain of p130Cas [15,30]. Additionally, the SH2 domains of Crk and CrkL can interact with other proteins that contain phosphotyrosine residues, such as paxillin (p70), and c-Cbl. The substrate domain also interacts with the SH2 domains of Src and Nck. These intricate interactions are influenced by activation states and strengths of binding partners and the particular cellular process in that cell type [10]. Considering the highly effective binding of p130Cas to SH2 domains, it is evident that the phosphotyrosine-containing p130Cas coexists with membranous or membrane-associated SH2-containing molecules, such as Src family kinases. The remaining p130Cas localizes in the cytoplasm as an unphosphorylated reserve [12]. The substrate domain of p130Cas underwent tyrosine phosphorylation by Src-family kinases, facilitating its interaction with CrkI, CrkII, CrkL, and Nck adaptors [2]. On the other hand, the N-terminal SH3 domains of Crk and CrkL interacted with proline-rich motifs found in several proteins, including C3G, DOCK180, and the Abl family [31]. The SH3 domain of p130Cas facilitated the interaction between p130Cas and various proteins, such as FAK, PYK2/RAFTK, FRNK kinases, PTP1B, PTP-PEST phosphatases, as well as other proteins, like C3G, CMS, CIZ, and vinculin. These interactions were involved in the recruitment and activation of Src-family kinases [2,32].

The Src-binding domain and the Cas family homology domain (CCHD) at the C-terminus of p130Cas are essential for enabling the binding of Src-family kinases and directing p130Cas to focal adhesions [2,33]. Elevated Src/FAK signaling triggered by Crk proteins resulted in the phosphorylation of paxillin and p130Cas, generating more binding sites for Crk proteins and intensifying the signaling pathway [16]. Additionally, p130Cas can be tyrosine-phosphorylated through protein kinase C activation independently of FAK and Src [34,35]. p130Cas and its homologs have the ability to interact with the Crk/CrkL SH2 domains following the phosphorylation of their substrate regions, leading to the recruitment of Crk/CrkL signaling pathway molecules to sites where focal adhesion assembly occurs. Upon the binding of p130Cas and its homologs to Crk family adaptors, proteins, such as C3G and SoS associated with focal adhesion complexes. However, upon the dephosphorylation of p130Cas, Crk switched its binding partner and bound to epidermal growth factor (EGF)-phosphorylated Cbl or paxillin [36]. It is possible that multiple tyrosine residues within the YxxP motifs are phosphorylated simultaneously, suggesting the potential existence of distinct molecular complexes involving p130Cas/Crk/DOCK180, p130Cas/Crk/C3G, p130Cas/Crk/Abl, p130Cas/Crk/c-Jun N-terminal kinase (JNK), and p130Cas/Crk/PI3K. However, experimental evidence is required to confirm this hypothesis [13]. Various effector proteins have been documented to either positively or negatively control the p130Cas-Crk/CrkL interaction. C3G, known as CrkSH3-binding guanine-nucleotide-releasing factor, serves as a guanine exchange factor for R-Ras, Rap1, and Rap2. Research has shown that C3G can directly bind to the SH3 domain of p130Cas and the p130Cas-interacting adaptor CrkI. Through activating its small GTPases, C3G exerted various effects on cytoskeletal remodeling, influencing adhesion and filopodia formation [37,38].

The non-receptor tyrosine kinase Abl plays a crucial role in regulating the coupling between p130Cas and Crk, leading to the inhibitory phosphorylation at Y207 of CrkL (Y221 of CrkII) and the subsequent uncoupling of the complex [1]. Upon the activation of growth factor receptors and integrins, Abl has been observed to localize to specific cellular areas where the p130Cas-Crk/CrkL assembly occurs, such as focal adhesions and membrane ruffles [1]. Further research has indicated that the inhibition of CrkII was primarily mediated by the proto-oncogene products Abl and Arg tyrosine kinases. These tyrosine kinases, initially identified in the Abelson murine leukemia virus (A-MuLV), play a direct role in the negative regulation of CrkII. Abl and Arg are the main tyrosine kinases responsible for suppressing CrkII (and CrkL) in cells. This role of Abl and Arg is evident from the fact that the phosphorylation of Crk Y221, a crucial marker, was nearly undetectable in Abl/Arg double knockout cells [13].

PTP-1B has been documented to dephosphorylate the inhibitory Y221 on CrkII, facilitating the binding of CrkII to YxxP motifs within the p130Cas substrate domain [1]. Additionally, p130Cas is a prominent target of the protein tyrosine phosphatase PEST (PTP-PEST). Elevated levels of PTP-PEST were associated with the decreased phosphorylation of p130Cas, diminished interaction between p130Cas and Crk, and hindered cell migration. The involvement of p130Cas in PTP-PEST-mediated focal adhesion turnover appears to revolve primarily around directing the localization of PTP-PEST to focal adhesions [39].

The formation of the p130Cas/Crk/DOCK180 complex was initiated by the phosphorylation of p130Cas by Src. Then, phosphorylated p130Cas interacted with Crk [40]. This interaction between p130Cas and Crk facilitated the recruitment of DOCK180, a Rac-GEF (Figure 2). The assembly of this complex resulted in the activation of Rac through conversion to Rac-GTP, leading to the stimulation of lamellipodia formation [41]. This complex played a crucial role in the recruitment and localization of Rac1 at the membrane, promoting actin cytoskeleton remodeling, pseudopodia extension, and focal adhesion turnover, ultimately enhancing cell migration. The degree of phosphorylation involved in this process was influenced by mechanical forces acting on the cell, which stretch the substrate binding domain and expose hidden tyrosine residues for phosphorylation [42,43]. Furthermore, the p130Cas-Crk complex became uncoupled, and p130Cas underwent cleavage by Caspase-3, which coincided with the delocalization of FAK from focal contact sites. Both the decoupling of p130Cas-Crk complexes and the cleavage of p130Cas were commonly observed during apoptosis. Consequently, disruptions in the p130Cas-Crk signaling seem to hinder the transmission of survival signals from the cytoskeleton [15,44].

p130Cas functions as a scaffold protein for various proteins containing SH2 and SH3 domains, and it forms a stable interaction with PYK2 through the SH3 domain of p130Cas and the C-terminal domain of PYK2. As Src phosphorylated the tyrosine residues of the YxxP motif of p130Cas, the phosphorylation levels of PYK2 or p130Cas were significantly decreased in osteoclasts lacking Src [45]. Also, Bmx/Etk, a constituent of the Tec/Btk family of non-receptor tyrosine kinases, plays a crucial role in regulating the phosphorylation of p130Cas. By binding to the C-terminal region of p130Cas, Bmx/Etk facilitated its phosphorylation and interaction with Crk, thereby augmenting membrane ruffling and haptotactic cell migration. This functional evidence establishes p130Cas as a direct target of Bmx/Etk in the context of actin cytoskeleton dynamics and cellular motility [46]. Moreover, urokinase-type plasminogen activator receptor (uPAR) promoted p130Cas phosphorylation and the p130Cas-CrkII assembly, suggesting a regulatory effect on various signaling pathways [47].

p130Cas has been recognized for its role as a mechanosensor, capable of detecting and reacting to mechanical stress. Studies have highlighted the significance of the substrate domain’s structure and phosphorylation in serving as transducers that convert physical mechanical stress into chemical responses. The p130Cas substrate domain typically adopts a compact folded structure, preventing the phosphorylation of its hidden tyrosine residues [32]. However, when subjected to mechanical stress, the substrate domain underwent stretching and unfolding, exposing the tyrosine residues within the domain. These exposed tyrosine residues were then phosphorylated by the SFKs [48]. Phosphorylation of the tyrosine residues in the substrate domain facilitated the interaction with signaling molecules like CrkII and C3G, subsequently activating various signaling pathways, such as Rap1, Rac1, and extracellular signal-regulated kinase (ERK) [49].

## 4. Functions of p130Cas and Crk Family Proteins in Normal Cells

The regulation of cell survival, proliferation, and motility in normal cells is influenced by the interaction of p130Cas with various proteins [50]. p130Cas, also known as BCAR1, is a versatile adaptor protein and crucial for embryonic development [3]. Additionally, p130Cas acts as a scaffold protein and possesses structural motifs that facilitate interactions with different signaling molecules, thereby regulating cellular processes, including cell adhesion, actin cytoskeleton organization, survival, proliferation, ECM degradation, and migration [11,51] (Table 1).

p130Cas is widely distributed in various tissues and is essential for embryonic development and actin filament assembly. Specifically, mouse embryos lacking p130Cas did not survive beyond 12.5 days due to severe issues related to the heart and circulation [3]. p130Cas-deficient embryonic fibroblasts exhibited disorganized actin filaments that were shorter in length. These cells also displayed defects in actin bundling and had defective focal adhesions that were small and poorly organized. Also, cells lacking p130Cas exhibited deficiencies in stress fiber formation, adhesion, actin bundling, and cell migration, all of which were rescued by the reintroduction of full-length p130Cas [51]. Notably, p130Cas expression was sufficient to enhance cell migration [8]. Depending on the sites of Src phosphorylation, p130Cas expression increased the rate of cell migration into a monolayer wound [40]. In addition, p130Cas mediated FAK-promoted cell migration [53]. While the role of p130Cas in motile cell types has been extensively studied, its importance in non-motile cell types, such as neurons, is also likely to be significant. Huang et al. demonstrated that the targeted loss of p130Cas in central nervous system neurons of Drosophila led to defects in neurite guidance and target fasciculation [68]. p130Cas is a crucial component in cell dynamics, acting as a molecule that assembles actin and stimulates integrins through surface receptors. This process is essential for extensive actin reorganization and cytoskeletal polarization, particularly in osteoclastic bone resorption. Nagai et al. used shRNA or a dominant-negative form of p130Cas to demonstrate significant hinderance in actin ring formation in vitro by the loss of p130Cas [45]. Furthermore, mice lacking p130Cas in osteoclasts displayed an osteopetrosis phenotype due to impaired bone resorption despite the presence of osteoclasts in the metaphyseal region, highlighting the vital role of p130Cas in facilitating actin dynamics and its impact on bone remodeling [45]. Normal human breast tissue expressed p130Cas mainly in the epithelial compartment. In the mouse mammary gland epithelium, p130Cas expression was regulated during development and was enriched in basal cells [69]. Recent research suggests that changes in p130Cas expression can affect mammary gland morphology and homeostasis [69]. Overexpression of p130Cas enhanced mammary branching morphogenesis in vivo during puberty, as well as in ex vivo mammary organoids stimulated with EGF or fibroblast growth factor (FGF). Overexpression of p130Cas in vivo in mice caused mammary hyperplasia and delayed involution via increasing proliferation and survival signaling [70].

In addition, p130Cas expression was found to markedly increase the phosphotyrosine content in both FAK and paxillin, thereby enhancing the invasiveness of Src-transformed cells significantly. This increased invasiveness was correlated with the heightened activation of matrix metalloproteinase-2 (MMP-2) and the formation of actin-rich podosomal aggregates. The abundant presence of tyrosine phosphorylation signaling in focal adhesions indicates that p130Cas plays a vital role as a scaffold in the formation of an invasive cell phenotype [52].

Crk and CrkL are also expressed widely in many types of cells and tissues. Matsuda et al. demonstrated that cell lines expressing Crk protein showed altered morphology [54]. Crk binding to Abl’s polyproline region prevents Crk phosphorylation, leading to increased migration [62]. In addition, Crk knockout suppresses PDGF-BB-inducible adhesion [57]. Also, Crk and CrkL are involved in various biological processes, such as Reelin-mediated neuronal migration [63], neuromuscular junction development [71], T cell adhesion and migration [56], kidney development and actin cytoskeletal remodeling [55], and natural killer cell expansion during mouse cytomegalovirus infection [61]. Loss of CrkL increased the apoptosis of neural crest cells [59]. Also, the loss of both Crk and CrkL in neural crest cells resulted in decreased proliferation and increased apoptosis with the defective differentiation of neural crest cells to vascular smooth muscle cells [58]. Park and Curran revealed that Crk and CrkL are crucial for preserving the structural integrity of the lens in post-natal eye development [72]. Park and Curran also showed that Crk and CrkL play crucial roles in fibroblast structure and motility by maintaining cytoskeletal integrity [31]. In the early mammalian embryonic development, Crk and CrkL controlled endothelial cell differentiation, angiogenesis, and vascular remodeling [60]. In addition, deletion of both Crk and CrkL hindered remodeling of the endocardial cushion and developmentally regulated the apoptosis of endocardial lineage cells [73].

The cooperative functions of p130Cas and Crk/CrkL have been reported in non-tumor cells. In mouse embryonic fibroblasts, Src family kinases-dependent targeting v-Crk to focal adhesions promoted the phosphorylation of p130Cas and FAK, enhancing cell migration [74]. Additionally, the presence of p130Cas, Crk, and DOCK180 at the filopodia tip complex was likely to promote filopodia formation when GD25b1B cells spread on invasin [65], instead of forming the typical adhesive focal complex structures containing FAK, vinculin, and paxillin. The presence of the p130Cas-CrkII complex was deemed necessary for this process. The expression of dominant-negative versions of p130Cas or CrkII inhibited the development of filopodia driven by β1B-integrin [65]. Also, Tamada et al. showed that in vitro results were consistent with in vivo observations of stretch-enhanced phosphotyrosine signals, accumulation of CrkII at cell-ECM contacts, and CrkII-p130Cas colocalization [66]. The importance of Src-dependent phosphorylation of p130Cas and its interaction with Crk in cell motility was highlighted by the observation that the knockdown of p130Cas and Crk using RNA interference significantly hindered Src-dependent lamellipodial extension and migration in epithelial cells [64]. Also, cell spreading was prevented by treating cells with siRNAs for p130Cas, Crk, and CrkL [64]. The leading lamellae of cells expressing either Crk or p130Cas displayed membrane ruffles rich in F-actin, giving them a characteristic motile phenotype. Remarkably, these membrane ruffles were home to both the Crk and p130Cas proteins. Additionally, the substrate domain of p130Cas and the SH2 domain of Crk and their complex were critical for the cell’s migratory properties [8]. Abl family kinases phosphorylate Crk at 221, decreasing Crk-p130Cas coupling, preventing cell migration, and inducing apoptosis [62]. Also, Kain et al. demonstrated that the transfection of COS-7 cells with a kinase-inactive form of c-Abl elevated CrkII-p130Cas coupling, improving cell migration [21].

p130Cas is believed to act as a facilitator of cell cycle initiation, contributing to cell transformation. The interaction between p130Cas and Crk was crucial for the activation of JNK, which in turn, drives the progression from G1 to S phase [67]. During mitosis, cells separated temporarily from the ECM until cytokinesis was finished, resulting in p130Cas phosphorylated on serine and threonine residues. Upon re-entering the G1 phase, serine/threonine phosphorylation decreased, presumably due to a PP2A-dependent mechanism. Simultaneously, normal levels of tyrosine phosphorylation were restored, suggesting that the equilibrium between serine/threonine and tyrosine phosphorylation may be modulated at various phases of the cell cycle [75]. Mechanical stretch allowed cells to detect and respond to mechanical pressures, enhancing tyrosine phosphorylation of p130Cas and its interaction with Crk while activating the small GTPase Rap1. Thus, the unfolding of p130Cas revealed effector binding sites and phosphorylation motifs, facilitating the conversion of external mechanical stimuli into internal biochemical signals [66].

Overall, the p130Cas protein is an important component of the cellular signaling network, regulating a variety of cellular functions. Its expression and activity are crucial for maintaining cell and tissue homeostasis [49]. Crk and its effector proteins are highly conserved throughout evolution and play significant roles in eukaryotic development [15]. Collectively, p130Cas, Crk, and CrkL, through the p130Cas-Crk/CrkL interaction in many cases, exhibit several essential functions, such as cell adhesion, migration, proliferation, survival, and development. Notably, dissociation of the p130Cas-Crk/CrkL complex hinders cell migration and causes apoptosis in normal cells. A comprehensive analysis is required to delineate the specific involvement of p130Cas or Crk/CrkL in these processes.

## 5. Functions of p130Cas and Crk Family Proteins in Tumor Cells

### 5.1. Expression of p130Cas, Crk, and CrkL in Tumor Cells

Due to its crucial function as a signal transducer, integrator, and scaffold in pathways regulating cell transformation, survival, motility and tumor progression in numerous cancer types, p130Cas has garnered significant attention in various aspects of cancer development and progression [1]. Crk family proteins have also been found to exhibit heightened expression in adenocarcinomas of the lung, breast, and stomach, as well as in sarcomas and glioma, as evidenced by immunohistochemical analyses and gene expression profiles [17,29]. The overexpression of Crk and CrkL in tumor cells triggered a significant tyrosine phosphorylation of scaffolding molecules, like p130Cas and paxillin, through Src family tyrosine kinases. This event, in turn, activated intracellular signaling pathways, leading to enhanced motility and increased aggressiveness of cancer cells [17] (Table 2).

#### 5.1.1. p130Cas Expression in Tumor Cells

p130Cas expression is increased in human breast carcinomas, indicating its potential involvement in cancer progression. Studies conducted on murine models have provided strong evidence supporting the role of p130Cas in the advancement of cancer. For instance, immunostaining analysis revealed the presence of p130Cas in atypical ductal hyperplasia specimens, while it was absent in normal tissues [70]. The overexpression of p130Cas in breast cancer cells has been shown to increase resistance to cytotoxic chemotherapies, including anti-estrogen agents and adriamycin. This finding suggests that p130Cas plays a role in activating survival pathways upon tyrosine phosphorylation. Also, p130Cas overexpression in these cells increased the percentage of cells in the S phase of the cell cycle [81]. The overexpression of p130Cas has been shown to impact the development of the mammary gland through the induction of epithelial cell hyperplasia and delayed involution. Moreover, the overexpression of p130Cas has been observed in a significant proportion of human breast cancers. Interaction of p130Cas with the HER2-Neu oncogene in pathological transformation indicates that p130Cas may play a critical role in the initiation of tumorigenesis. These findings are further supported by the activation of signaling pathways by p130Cas, leading to enhanced proliferation and reduced apoptosis. Conversely, the depletion of p130Cas using siRNA has been found to increase apoptosis by suppressing survival signaling pathways in transformed epithelial cells [70]. The outcomes of RNA interference experiments suggest that p130Cas is involved in the progression of cancer [50]. An increased level of tyrosine phosphorylation of p130Cas is a notable characteristic of mesenchymal-like estrogen receptor-negative ER- breast cancer cell lines. Knockdown and rescue of p130Cas has provided insights that this signaling event is integral to the processes of cell proliferation, survival, and the assembly of F-actin, and encompasses the stress fibers related to focal adhesions in the BT-549 cell line [77]. Overexpression of CASP8 and FADD-like apoptosis regulator (CFLAR) increased p130Cas phosphorylation, leading to an increase in F-actin polymerization and the development of elongated pseudopodia in A549 and MCF7 cells. Notably, the spreading areas of MCF7-CFLAR and MCF7-DED cells were significantly diminished when treated with p130Cas siRNAs [76]. Additionally, the lack of p130Cas was associated with enhanced nuclear localization of Smad2/3 and a reduction in the proliferation rates of metastatic mammary epithelial cells [78].

In lung cancer tissues, the expression of POLR2A and p130Cas was significantly higher than matched normal tissues. A strong positive correlation was identified between elevated POLR2A levels and the overexpression of p130Cas. Following the knockout of p130Cas in H1975 and H1299 cells, a significant decrease in POLR2A expression was recorded. Moreover, the proliferation of H1975 and H1299 cells was significantly hindered after the p130Cas knockout [79]. A high level of p130Cas expression in circulating tumor cells was correlated with the expression of CD274 and the occurrence of EMT. Additionally, the protein Rac1, in partnership with p130Cas, was recognized as playing a crucial role in the development of lung adenocarcinoma. The collaboration between Rac1 and p130Cas was instrumental in enhancing cell proliferation and provided resistance to anoikis in lung adenocarcinoma cells [80].

An analysis of human prostate cancer samples has revealed a positive correlation between increased p130Cas expression and higher-grade tumors. Specifically, 15% of low-grade localized tumors expressed p130Cas, while 48% of high-grade tumors and 80% of Castration-resistant prostate cancers exhibited p130Cas expression [126]. The expression of p130Cas was found to be linked to elevated levels of epidermal growth factor receptor (EGFR) and the reduced expression of the metastasis suppressor Cluster of Differentiation 82 (CD82/KAI1) [126]. Likewise, 76% of ovarian cancer patient samples exhibited heightened levels of p130Cas, and higher levels of this protein were associated with a more advanced stage of the disease and lower survival rates [82]. Also, the expression of p130Cas in hepatocellular carcinoma has a correlation with decreased levels of E-cadherin and beta-catenin expression, as well as poorer pathohistological grades and prognosis. It is worth noting that the abnormal expression of beta-catenin and reduced expression of E-cadherin are correlated with the positive expression of p130Cas. Hepatocellular carcinoma patients who exhibited positive expression of p130Cas were at a higher risk of developing lymph node metastasis. These findings lead to intriguing speculation that the overexpression of p130Cas in hepatocellular carcinoma may disrupt the stability of the cadherin/catenin complex [127]. p130Cas was instrumental in driving an invasive cellular phenotype in Src-expressing cells. This effect was achieved through the activation of MMP-2 and the upregulation of actin polymerization processes that contributed to the assembly of podosomes and the extension of the plasma membrane [52].

#### 5.1.2. Crk and CrkL Expression in Tumor Cells

Although the control and Crk knockdown cells exhibited paxillin-positive adhesions, the Crk knockdown breast cancer cells demonstrated a significant delay in cellular spreading, suggesting that lower levels of Crk expression negatively impacted integrin-mediated spreading [94]. In the MDA-MB-435 cell line, the overexpression of CrkL enhanced cell proliferation, while the application of small interfering RNA to knockdown CrkL in the MDA-MB-453 cell line led to a decrease in cell proliferation. The investigation into cell cycle-related molecules indicated that CrkL enhanced the expression levels of cyclin D1 and phosphorylated extracellular signal-regulated kinase in the MDA-MB-435 cell line [100]. Also, in a murine model of spontaneous metastasis involving 4T1 breast adenocarcinoma in immune-competent BALB/c mice, the genetic deletion of Crk via CRISPR-Cas9 technology resulted in an increase in populations of anti-tumor immune cells, as well as elevated levels of cytotoxic effector cells and immune surveillance cytokines within the primary tumor. This genetic modification was associated with a marked decrease in the proliferation of tumor cells [101].

The glioblastoma KMG4 cell lines with Crk knockdown via siRNA exhibited a notable reduction in their initial adhesion to laminin. Furthermore, these cells demonstrated a suppression of cell proliferation [93]. In addition, using siRNAs to achieve CrkL knockdown in the U-118MG human glioblastoma cell line resulted in a notable reduction in cell size, alongside a marked inhibition of cell proliferation and adhesion. In contrast, the concurrent knockdown of both Crk and CrkL elicited even more pronounced morphological changes and a stronger inhibition of both proliferation and adhesion [87]. Further, Crk Y251F glioblastoma cells displayed a more flattened morphology, which was associated with an early decline in cell spreading on fibronectin-coated dishes [86].

The overexpression of CrkL significantly promoted cell proliferation within CaSki cervical carcinoma cell lines. This enhancement was linked to heightened phosphorylation levels of Src and Akt [97]. Also, miR-429 appears to play a role in the suppression of tumor proliferation by targeting the ZEB1 and CrkL signaling pathways. Furthermore, the use of siRNA to silence ZEB1 and CrkL has been shown to decrease apoptosis, which corroborates the effects observed with miR-429. This finding suggests that ZEB1 and CrkL are significant contributors to the inhibition of cervical cancer advancement through apoptotic processes [98]. Also, the expression levels of CrkL were predominantly increased in samples of stage 1 cervical cancer, and the silencing of CrkL resulted in a decrease in cell proliferation [99].

In lung carcinomas, the expression of the CrkL protein was significantly elevated relative to that in adjacent normal lung tissue. The overexpression of CrkL in the HBE and H1299 cell lines associated with lung carcinomas stimulated cell proliferation by advancing cell cycle progression. A further examination of molecules related to the cell cycle indicated that CrkL was responsible for the induction of cyclin D1 and cyclin B1 expression, as well as an increase in the phosphorylation of Rb [104]. In the context of non-small cell lung cancer, the Src Homology domains of CrkI were overexpressed in the H157, Rh2, and A549 cell lines. Notably, the expression of the Crk-SH3N domain led to the promotion of epithelial morphology in H157 cells and further improved the epithelial morphology in A549 and Rh2 cells, relative to cells that were transfected with either the CrkSH2 domain or an empty vector [88].

A study by Watanabe et al. showed that hepatocyte growth factor (HGF) significantly enhanced the motility and dispersal of synovial sarcoma cell lines, which correlated with the substantial activation of Rac1, extensive formation of filopodia, and membrane ruffling. Notably, the removal of Crk in these cells led to a disruption of the actin cytoskeleton organization [92]. Also, the RNA interference-mediated depletion of Crk substantially hindered the proliferation of the synovial sarcoma cell lines HS-SYII, SYO-1, and Fuji. The authors demonstrated augmented transcriptional activity and a significant increase in the expression of the p16INK4A gene, which caused cell cycle arrest at the G1 phase [92].

The reduction in Crk levels markedly inhibited the proliferation of bladder cancer cell lines, 5637 and UM-UC-3, and the results correlated with diminished ERK activity [109]. Inhibition of CrkI/II expression led to a reduction in cell spreading on the ECM with a decrease in actin stress fibers and a lower incidence of mature focal adhesion formation [90].

The proliferation of head and neck squamous cell carcinoma cells, HSC-3 and HSC-4, significantly decreased in the presence of CrkL knockdown relative to the control group. The attachment of cells to dishes coated with fibronectin and collagen was markedly reduced by CrkL knockdown in HSC-3 cells. Additionally, immunofluorescence staining indicated a decrease in focal adhesion formation in these CrkL knockdown HSC-3 cells [95]. CrkL exhibited consistent and elevated expression levels in both rhabdomyosarcoma cell lines and tumor tissues. The presence of CrkL was essential for the proliferation of both alveolar and embryonal subtypes of rhabdomyosarcoma [106]. The depletion of CrkL in SGC-7901 gastric cancer cells led to a significant decrease in cell proliferation, as well as a pronounced interruption of the cell cycle at the G0/G1 phase [107]. The overexpression of CrkL, achieved through the transfection of a plasmid into Ishikawa cells derived from human endometrial carcinoma, significantly stimulated cell proliferation and advanced cell cycle progression. Additionally, the elevated levels of CrkL were associated with a decrease in apoptosis among Ishikawa cells exposed to cisplatin and diminished cleavage of caspase-3 and caspase-9 [108]. Analyses of gene profiling have revealed that CrkL was a likely downstream target of the miR–215–PCAT-1 axis in hepatocellular carcinoma. The suppression of CrkL expression led to a significant reduction in cell proliferation [103].

A significant reduction in cell proliferation, chemoresistance, as well as cells forming dense and three-dimensional clusters were observed in colorectal cancer cells deficient in Crk and CrkL, and the results correlated with decreased levels of ERK1/2 phosphorylation and c-Myc protein. Mechanistically, Crk and CrkL were found to play a critical role as amplifiers of Src/FAK signaling at focal adhesions, operating through a novel positive feedback loop that was dependent on Rap1 [16].

#### 5.1.3. Cooperative Roles of p130Cas and Crk/CrkL Overexpression in Tumor Cells

In hematopoietic cells transformed by the Bcr/Abl chimeric tyrosine kinase, p130Cas is known to create a signaling complex with CrkL. This chimeric tyrosine kinase is a result of reciprocal translocation between chromosomes 9 and 22. Bcr/Abl is the primary cause of chronic myelogenous leukemia (CML) and Philadelphia chromosome-positive acute lymphoblastic leukemia (Ph+ ALL). The study of Bcr/Abl expressing cell lines and samples from CML and Ph+ ALL patients has shown that Bcr/Abl is associated with CrkL and p130Cas [38,128]. In Bcr/Abl-transformed cells, the disrupted interaction between p130Cas and the focal adhesion protein tensin resulted in the adhesion abnormalities typical of CML cells [128]. Additionally, the Rap1 GEF, C3G, forms Bcr/Abl-CrkI-C3G-p130Cas complexes by binding constitutively to Crkl, with subsequent phosphorylation facilitated by Rap1 [38].

Overall, in various types of cancer, such as breast, prostate, ovarian, lung, colorectal, pancreatic, and hepatocellular carcinoma, as well as in glioma, melanoma, anaplastic large cell lymphoma, and chronic myelogenous leukemia, there has been an increase in the expression of p130Cas and Crk/CrkL. Conversely, reducing the expression levels of those proteins in breast, prostate, and ovarian cancer has been shown to impede tumor growth and the advancement of cancer cells [11]. Furthermore, p130Cas, Crk, CrkL, and the p130Cas-Crk/CrkL complex play a significant role in tumor-specific cellular functions, such as cell transformation, migration, and invasion, as elaborated upon in the subsequent discussion.

### 5.2. Cellular Transformation

#### 5.2.1. Contribution of p130Cas to Cellular Transformation

p130Cas plays an important role in cellular transformation. Increased POLR2A levels have been shown to have a tight correlation with p130Cas overexpression. Following the knockout of p130Cas in lung cancer cells, including H1975 and H1299 cells, a significant decrease in POLR2A expression was observed. The knockout of p130Cas resulted in a notable decrease in the colony formation capacity of H1975 cells [79]. Moreover, Rac1, alongside p130Cas, was determined to be pivotal in the carcinogenic mechanisms underlying lung adenocarcinoma. The interaction between Rac1 and p130Cas was essential for colony formation in those cells [80]. p130Cas has also been also demonstrated to play a role in Src-mediated transformation. When reintroduced into p130Cas-deficient mouse embryonic fibroblasts, p130Cas increased the phosphorylation of FAK and paxillin, indicating its ability to enhance oncogenic Src activity [52].

#### 5.2.2. Contribution of Crk and CrkL to Cellular Transformation

Crk knockdown in human ovarian cancer cell lines led to a decrease in colony formation in medium-sized cultures and notable morphological changes [89]. Additionally, in the SKOV3 ovarian cancer cell line, the suppression of Crk expression was associated with a notable decrease in cell invasion. Furthermore, this knockdown caused a significant reduction in the quantity of medium-sized colonies and modified the morphology of the actin cytoskeleton [91]. The knockdown of Crk significantly diminished the growth characteristics of glioblastoma KMG4 cells as well as their ability to form colonies and propagate in xenograft experiments [93]. Also, the expression of Crk Y239F within breast cancer cells led to a delay in the activation of Src kinase and a reduction in cell transformation [57]. Crk-silenced SYO-1 synovial sarcoma cells demonstrated a marked decline in proliferation and were entirely incapable of colony formation in soft agar [105]. The reason behind the high transforming potential of CrkI appears to be its structural resemblance to v-Crk. In contrast, CrkII and CrkL exhibit little to no transforming activity due to the presence of a regulatory phosphorylation site at their C-terminal region [129]. In vitro studies demonstrated that CrkII shRNA markedly inhibited colony formation in prostate cancer cells, yet it did not lead to a significant reduction in tumor volume [112]. Crk knockdown in pancreatic cancer cells, which were established through the application of siRNA in PANC-1, AsPC-1, and MIA PaCa-2 cell lines, led to a notable decrease in cell adhesion. Furthermore, there was a marked reduction in the number of large colonies formed by Crk knockdown cells in both the PANC-1 and AsPC-1 lines [96].

CrkL has been identified as a factor associated with the malignant behavior of Hca-P, a murine hepatocellular carcinoma cell line with a lymph node metastatic rate of 25%. CrkL overexpression in Hca-P significantly improved its malignant biological characteristics. Furthermore, this overexpression was found to substantially inhibit the proliferation and colony formation abilities of the Hca-P cells [102]. The consistent overexpression of CrkII led to a notable enhancement in the colony-forming efficiency of Hca-P cells [110]. In addition, colony formation in hepatocellular carcinoma cells reinforced the oncogenic characteristics attributed to CrkL, and the elevated levels of CrkL counteracted the tumor-suppressive functions of miR-215 [103]. Also, the HBE and H1299 cell lines of lung carcinomas showed a notable rise in colony formation associated with CrkL overexpression, compared to the controls [104].

In cervical cancer, miR-429 played a role in reducing colony formation via the ZEB1 and CrkL signaling pathways [98]. The transfection of human endometrial carcinoma Ishikawa cells with a plasmid encoding CrkL resulted in an increased capacity for colony formation among the transfected cells [108]. SASH1, which contains a Src-homology 3 domain, inhibited EMT by engaging with the oncoprotein CrkL in colorectal cancer cells. This interaction prevented the CrkL-mediated activation of Src kinase, which was fundamentally required for the EMT process. Also, a significant reduction in both the quantity and size of colonies was observed in cells deficient in SASH1 when CrkL was absent [111].

Overexpression of CrkL in fibroblasts also resulted in transformation [113]. Koptyra et al. showed that Crk and CrkL were both essential for v-Fos-induced cellular transformation in fibroblasts, while Crk was more important than CrkL in v-Ras-mediated transformation. Furthermore, both Crk-null and CrkL-null fibroblasts transformed by v-Fos showed a decrease in the phosphorylation of p130Cas with the effect being more pronounced in Crk-null cells. Their results also suggest that during v-Fos transformation, Crk is primarily involved in mediating the tyrosine phosphorylation of cellular proteins, including p130Cas [114].

#### 5.2.3. Cooperation Between p130Cas and Crk/CrkL for Cellular Transformation

Elevated levels of tyrosine phosphorylated p130Cas in numerous tumor cells suggest a crucial role of p130Cas in cellular transformation [13]. p130Cas was identified as a heavily tyrosine phosphorylated substrate in cells transformed by Src and Crk [130]. The significance of p130Cas in Src and Crk transformation is underscored not only by its stable and persistent interaction with SFKs but also by findings indicating that tyrosine kinases and Ras oncogenes were unable to transform p130Cas-deficient fibroblasts [131]. It is worth noting that a fusion protein comprising the p130Cas substrate domain and a modified Src kinase domain, which is consistently phosphorylated, acted as a dominant-negative regulator of v-Crk transformation and inhibited JNK activation. This finding provides further support for the involvement of JNK in p130Cas-mediated transformation [123]. Moreover, the expression of CrkI in glioblastoma U87MG cells and tyrosine phosphorylation of p130Cas induced transformation [25]. Taken together, cellular transformation is likely to be contributed to by the shared function of p130Cas, Crk, and CrkL.

### 5.3. Cell Motility and Migration

#### 5.3.1. Contribution of p130Cas to Tumor Cell Motility and Migration

p130Cas is essential for cell motility by modulating the activity of small GTPases, which are key regulators of actin cytoskeleton dynamics. To achieve coordinated and persistent movement, cells must have precise spatiotemporal control of signaling events, which is necessary during cell migration. A typical mode of movement in mesenchymal cells involves a delicate balance between the protrusion of the cell membrane in the forward direction and the retraction of the tail through the contraction and release of focal contacts. This process requires an asymmetric organization of the assembly and disassembly of focal adhesions and stress fibers [1]. RNA interference to attenuate p130Cas expression and restoration of wild-type p130Cas were used to show that p130Cas is a pivotal signaling molecule in the migration processes of estrogen receptor-negative breast cancer cells, BT-549 [77]. Also, the silencing of p130Cas in ovarian carcinoma led to statistically significant reductions in tumor cell migration [82].

When p130Cas was unphosphorylated, it was distributed within the cytosol. However, when it underwent tyrosine phosphorylation in response to ligand stimulation, p130Cas was translocated to the cell membrane [12]. Various growth factors and hormones, such as FGF-2, VEGF, M-CSF, endothelin, angiotensin, and MIP-3a, have been found to increase p130Cas tyrosine phosphorylation, which is linked to cell motility and invasion [50]. Additionally, p130Cas has been observed to colocalize with various focal adhesion-associated proteins, such as Src, FAK, PYK2, Nck, and Crk within focal adhesions [1]. The presence of the SH3 and carboxy-terminal domains in p130Cas was necessary for its localization to focal adhesions [4].

The chemotactic migration process was influenced by the presence of p130Cas and its interaction with ligand-induced Src-mediated tyrosine phosphorylation. This interaction led to the assembly of a complex involving p130Cas, Crk, DOCK180, and ELMO at focal adhesion sites. This complex was considered an unconventional GEF and played a crucial role in the localized activation of the small GTPase Rac [132,133]. The activation of Rac, in turn, promoted actin polymerization, which was necessary for the formation of lamellipodia and membrane ruffles [134]. The actin-related protein 2/3 (ARP2/3) and p21-activated kinase (PAK) were activated by Rac and contributed to cell migration [135]. Rac1, in conjunction with p130Cas, was identified as a significant contributor to the carcinogenic processes in lung adenocarcinoma. Together, Rac1 and p130Cas facilitated migration in lung adenocarcinoma cells [80]. CFLAR interacted with p130Cas and facilitated the phosphorylation of p130Cas through its DEDs domain, consequently boosting cell migration [76]. Additionally, p130Cas could bind to different protein motifs of several phosphatases and kinases, such as FAK and Src. This discovery sheds light on a new mechanism involving CFLAR in cell migration through the CFLAR-p130Cas interaction. It is worth noting that CFLAR, an analog of Caspase 8, is associated with cell migration, similar to Caspase 8, which has been linked to migration in both normal and tumor cells [76,136]. The activation and ligation of the EGFR resulted in the Src-dependent phosphorylation of two vital tyrosine residues within p130Cas. This modification led to the formation of a Cas/Nck1 complex, which promoted Rap1 signaling. The GTP loading of Rap1 played a crucial role in the migration of pancreatic carcinoma cells [83].

#### 5.3.2. Contribution of Crk and CrkL to Tumor Cell Motility and Migration

Inhibition of Crk expression resulted in the diminished motility of KMG4 glioblastoma cells, causing a notable delay in the wound recovery [93]. In addition, cells overexpressing Crk Y251F exhibited a lower migration rate [86]. Moreover, the expression of CrkL was observed in both U87 and U251 cell lines, with its activation mediated by transforming growth factor-beta 1 (TGF-β1). Notably, knockdown of CrkL resulted in a marked decrease in the levels of phospho-ERK1/2 and MMP9 and an increase in phospho-Smad2 expression relative to the control. In contrast, the overexpression of CrkL led to an increase in phospho-ERK1/2 and MMP9 levels, accompanied by a decrease in phospho-Smad2. Additionally, the knockdown of CrkL significantly influenced the migration of U87 and U251 glioblastoma cells in response to TGF-β signaling [115]. Also, the double knockdown of Crk and CrkL completely blocked the migration and invasion of U-118MG human glioblastoma cells, an effect that could be mitigated by the transient overexpression of CrkL, but not Crk [87].

In samples from colorectal cancer patients, a strong correlation was noted between the expression of the Crk family proteins and the EMT regulator ZEB1, particularly in regions characterized by invasiveness. The loss of either Crk or CrkL resulted in a marked decrease in both cell migration and invasion [16]. Also, the augmented migration of cells lacking SASH1 was fully reversed by the elimination of CrkL. Therefore, the EMT instigated by the absence of SASH1 is entirely reliant on the interaction with CrkL [111].

In bladder cancer cell lines 5637 and UM-UC-3, silencing of Crk was associated with a significant reduction in cell migration. It is important to highlight that the depletion of Crk resulted in the diminished phosphorylation of c-Met and the downstream scaffold protein Gab1, occurring in both hepatocyte growth factor-dependent and independent contexts [109]. The introduction of wild-type paxillin or Crk in excess effectively counteracted the migration-deficient phenotype present in Nara Bladder Tumor II cells. It has been demonstrated that the SH2 and SH3 domains of CrkII are crucial for enabling migration that is mediated by collagen [116].

The suppression of CrkL in SKOV-3 cells resulted in a significant reduction in the CCL19-induced expression of p-ERK and various EMT-related biomarkers, such as N-cadherin, Snail, and MMP9, relative to the control conditions. In contrast, there was no observable change in the expression levels of p-Akt. Additionally, functional analyses indicated that the knockdown of CrkL substantially impaired the migration and invasive potential of SKOV-3 cells [117]. Also, the application of RNA interference to achieve Crk knockdown in a human ovarian cancer cell line resulted in a significant reduction in cell motility [89].

The overexpression of CrkL in murine hepatocarcinoma Hca-P cells led to a decrease in migration capacities [102]. Also, the in vitro migration and invasion potential of Hca-P cells increased by approximately 179% and 156%, respectively, following the stable elevation of CrkII levels [110].

In cervical cancer cell lines, ZEB1 and CrkL expression levels were found to be inversely correlated to the levels of miR-429. Additionally, the specific knockdown and overexpression of these genes replicated the functional outcomes associated with miR-429 overexpression and the inhibition of cell migration [98]. Further, the expression of Y239F Crk in Crk-null mouse embryonic fibroblasts, glioma and breast cancer led to a marked reduction in the speed of cell migration compared to the reconstitution with wild-type Crk [57].

RNA interference-mediated knockdown of CrkI/II produced a substantial decline in the migration of several malignant and metastatic basal breast and other human cancer cell lines, such as MDA-MB-231, MDA-MB-435S, H1299, KB, and HeLa [90,94].

The sustained phosphorylation of the c-Met-docking protein Grb2-associated binder 1 (Gab1) in response to HGF required the presence of the Crk adaptor protein. This mechanism contributed to the increased motility observed in human synovial sarcoma cell lines, including SYO-1, HS-SY-II, and Fuji. When Crk was eliminated from these cells, there was a total cessation of HGF-induced Rac1 activation and a marked decrease in cell motility [92]. Also, knockdown of Crk impaired the motility of pancreatic ductal adenocarcinoma (PDAC) cells by lowering the phosphorylation levels of c-Met [96].

Suppressing CrkII expression via RNA interference resulted in a significant reduction in the migration of the OSC20 oral squamous cell carcinoma cell line. Additionally, this downregulation correlated with the decreased expression of DOCK180, p130Cas, Rac1, and the actin-associated scaffolding protein cortactin [118].

CrkL knockdown in head and neck squamous cell carcinoma cells, HSC-3 and HSC-4, resulted in a notable reduction in cell motility. The HSC-3 cells with CrkL knockdown exhibited lower levels of active Rap1 in comparison to the control cells [95]. A significant reduction in the motility of non-small cell lung cancer cells, including A549 and H157, was observed with the expression of the Crk-SH3N domain [88]. On the other hand, CrkL played a crucial role in mediating the EMT induced by the CCL20/CCR6 axis through the Akt signaling pathway, rather than the Erk1/2 pathway during gastric cancer development. This finding suggests that the CCL20/CCR6–CrkL–Erk1/2–EMT signaling pathway could serve as a potential target for therapeutic intervention to inhibit the progression of gastric cancer. Furthermore, the silencing of CrkL resulted in a reduction in migration and invasion in MGC803 cells [119]. The silencing of CrkII through shRNA-mediated knockdown in prostate cancer cells markedly reduced the processes of cancer cell migration [112].

#### 5.3.3. Cooperation Between p130Cas and Crk/CrkL for Tumor Cell Motility and Migration

The interaction between the adaptor protein Crk and the docking protein p130Cas plays a crucial role in regulating cell migration in various types of invasive cancer cells [17,124]. The coupling between p130Cas and Crk is believed to function as a “molecular switch”, and this interaction leads to the recruitment of multiple proteins, triggering cell migration and potentially enhancing the invasiveness of certain tumor cells [8]. It is worth noting that ErbB2 facilitated the coupling of p130Cas and c-CrkII, resulting in migration regulation [125]. Also, CrkI expression stimulated cell migration by activating the FAK/p130Cas/CrkI/DOCK180 pathway [25]. The tyrosine phosphatase PTP-1B dephosphorylated Y221 of Crk, facilitating the coupling of p130Cas and Crk, thereby promoting cell migration in fibrosarcoma. Additionally, phosphatases may also exert their influence on p130Cas itself [18]. Therefore, the molecular interaction between Crk and p130Cas acts as a regulatory switch, controlling cell migration in prostate cancer cells [124]. In contrast, mutants lacking the YDxP motifs in the substrate domain and the C-terminal Src-binding domain failed to restore the migratory behavior, likely due to their inability to bind Crk and interact with Src kinase [40,64]. Taken together, tumor cell migration, a crucial cancer trait, can occur through the formation of the p130Cas-Crk/CrkL complex. It is likely that the disassociation of the p130Cas-Crk/CrkL complex results in decreased cell migration, whereas association of the p130Cas-Crk/CrkL complex promotes cell migration.

### 5.4. Invasion and Metastasis

#### 5.4.1. Contribution of p130Cas to Invasion and Metastasis

The ability of tumor cells to move is crucial for their invasion into the neighboring tissue and is a fundamental prerequisite for metastasis. The metastatic process initiates with the infiltration of primary tumor cells into the surrounding tissue, followed by their entry into blood and lymphatic vessels, circulation throughout the body, and eventual arrest in distant capillary beds, leading to extravasation into new locations [137,138]. Matrix metalloproteinase [MMP] expression plays a vital role in invasion by facilitating the degradation of the ECM, allowing cell invasion into neighboring tissues [139]. The abnormal expression of p130Cas proteins, whether through gene amplification, transcriptional upregulation, or changes in protein stability, along with subsequent phosphorylation, has been strongly associated with a negative prognosis, increased cell invasion, and metastasis in cancer. Additionally, this abnormal expression has been linked to resistance to initial chemotherapeutic treatments in various types of tumors, such as breast and lung cancers, glioblastoma, and melanoma [17]. The activation of MMPs after translation has also been associated with p130Cas. The degradation of the ECM at focal adhesion sites was facilitated by MT1-MMP in various cancer cell lines. This process necessitated the presence of a p130Cas-FAK complex for the proper recruitment of MT1-MMP. The activity of MT1-MMP was regulated through phosphorylation mediated by Src. Furthermore, the disruption of the FAK-p130Cas-MT1-MMP complex was observed to decrease ECM degradation and invasion [85].

The results with reduction in p130Cas expression in estrogen receptor-negative breast cancer cell lines, like BT-549 cells, by RNA interference, and contrasting effects between the wild-type p130Cas and a signaling-deficient variant suggested that tyrosine phosphorylation of p130Cas is a significant signaling event for invasion [77]. Also, a significant increase in Smad2/3 activation was observed upon the depletion of p130Cas expression in TbR-II-expressing metastatic mammary epithelial cells, which counteracted the cellular invasion and early dissemination of mammary tumor cells prompted by TGF-β [78]. The co-occurrence of p130Cas overexpression and ErbB2 activation resulted in increased activities of the PI3K/Akt and Erk1/2 MAPK signaling pathways and the increased invasion of mammary acini. The mechanisms of invasion were mediated by distinct downstream effectors, with mTOR/p70S6K being activated through Erk1/2 MAPK signaling and Rac1 being activated via PI3K/Akt signaling [84]. Rac1, working in tandem with p130Cas, was found to be a key player in the carcinogenesis of lung adenocarcinoma. This collaboration between Rac1 and p130Cas was crucial for the induction of invasion in lung adenocarcinoma cells [80]. The silencing of p130Cas in ovarian carcinoma also led to a significant decrease in tumor cell invasion [82].

#### 5.4.2. Contribution of Crk and CrkL to Invasion and Metastasis

CrkL expression was detected in U87 and U251 glioblastoma cell lines, and CrkL was activated by transforming growth factor-beta 1 (TGF-β1). The suppression of CrkL expression resulted in a significant decline in phospho-ERK1/2 and MMP9 levels, while phospho-Smad2 expression was notably elevated compared to the control. In contrast, the overexpression of CrkL led to increased levels of phospho-ERK1/2 and MMP9, while phospho-Smad2 levels decreased. Moreover, knockdown of CrkL had a significant effect on the invasion of U87 and U251 cells induced by the TGF-β pathway [115]. In HS683 glioma cells and breast cancer, the presence of Crk Y239F inhibited the activation of Src kinase and diminished the invasive capabilities of the cells [57]. Also, the phosphorylation of Crk at Tyr251 enhanced the invasive behavior of glioma cells. At the molecular level, the phosphorylation at Tyr251 was negatively influenced by Abi1, which competes with Crk for binding to Abl, thereby reducing Abl’s transactivation. Thus, Crk and Abi1 have been proposed to play opposing biological functions and serve as a molecular rheostat that regulates Abl activation and cell invasion [86].

A549 cells, a representative of non-small cell lung cancer, showed enhanced aggressive behavior when the phosphomimetic mutant of CrkII at serine 41 was expressed. Conversely, the expression of a phosphodeficient mutant of CrkII at this site led to diminished invasive potential. PAK1 has been identified as a key mediator of serine phosphorylation of CrkII. Silencing PAK1 resulted in elevated levels of p120-catenin in both A549 and H157 cell lines. Moreover, the reduction in PAK1 expression corresponded with a decrease in the invasive characteristics of A549 cells [88,120]. In addition, the induction of EMT in A549 human lung adenocarcinoma cells by Crk was mediated through the distinct regulation of the Rac1/Snail and RhoA/Slug signaling pathways, which led to a decrease in E-cadherin expression alongside increased levels of N-cadherin, fibronectin, and MMP2 [122].

Silencing of CrkI/II through RNA interference led to a marked reduction in the invasion of various malignant and metastatic basal breast and other human cancer cell lines, including MDA-MB-231, MDA-MB-435s, H1299, KB, and HeLa [90,94]. Also, in murine 4T1 breast adenocarcinoma, Crk knockout effectively diminished both EMT and PD-L1 expression, leading to an additive effect alongside anti-PD1 therapy in curtailing tumor growth [101].

The expression levels of ZEB1 and CrkL exhibited an inverse correlation with miR-429 levels in cervical cancer cell lines. Furthermore, the individual knockdown and overexpression of these target genes mirrored the effects observed with miR-429 overexpression and the suppression of cellular invasion [98]. Also, the administration of the Src inhibitor dasatinib counteracted the effects of CrkL on cell invasion [97]. Using RNA interference to induce Crk knockdown in a human ovarian cancer cell line demonstrated a marked suppression of invasion [89]. Also, the inhibition of CrkII expression through RNA interference led to a diminished invasion capacity in the oral squamous cell carcinoma cell line OSC20. Furthermore, the downregulation of CrkII was associated with a decrease in the levels of DOCK180, p130Cas, Rac1, and the actin-binding scaffold protein cortactin [118].

An increase in miR-126 expression was found to significantly lower the protein levels of Crk, which has been established as a direct target of this microRNA. Furthermore, knockdown of Crk notably diminished the invasive potential of gastric cancer cells [121]. The Crk knockdown in the bladder cancer cell lines 5637 and UM-UC-3 demonstrated a substantial decrease in cell invasion. Notably, the removal of Crk was linked to a reduction in the phosphorylation of c-Met and the downstream scaffold protein Gab1, which occurred regardless of the presence of hepatocyte growth factor [109]. Following the overexpression of CrkL, there was an observable reduction in the invasion capacities of murine hepatocarcinoma Hca-P cells [102]. Moreover, the increased invasion of SASH1-deficient colorectal cancer cells was completely counteracted by the removal of CrkL. Thus, the EMT that occurs due to the loss of SASH1 is fundamentally dependent on the presence of its interaction partner, CrkL [111].

#### 5.4.3. Cooperation Between p130Cas and Crk/CrkL for Invasion and Metastasis

In prostate cancer cells, the knockdown of CrkII via shRNA-mediated silencing led to a substantial inhibition of cancer cell invasion [112]. In addition, the expression level of p130Cas was found to be associated with the metastatic potential of prostate cancer cell lines. Western blot analysis revealed that highly invasive PC-3M cells exhibited the highest levels of p130Cas protein, while moderately invasive DU-145 cells displayed the lowest levels. These findings suggest that p130Cas plays a role in the invasion of prostate cancer cells. Knockdown of p130Cas in PC-3M cells resulted in a significant decrease in both cell migration and invasion, similar to the effects observed with CrkI knockdown. Moreover, co-immunoprecipitation assays revealed that p130Cas interacts with CrkI in prostate cancer cells, and the stability of both proteins is mutually dependent, potentially facilitated by their interaction. Collectively, these results indicate that the interaction between CrkI and p130Cas plays a crucial role in promoting the migration and invasion of prostate cancer cells [124].

In ErbB2-transformed cells, p130Cas formed a crucial component of a quaternary complex along with ErbB2, Src, and FAK. This complex has been shown to upregulate the activity of Src in breast cancer. Additionally, ErbB2 has been found to enhance the coupling between p130Cas and CrkII, leading to increased activation of ERK and subsequent invasiveness [125]. Also, the expression of CrkI in U87MG cells enhanced invasiveness through the activation of the FAK/p130Cas/Crk/DOCK180 pathway [25]. Notably, the signaling pathways involving uPAR and the p130Cas-Crk complex are similar. It has been observed that both p130Cas and Crk are necessary for the activation of Rac by uPAR and for the invasion of colon cancer cells expressing uPAR [47]. The phosphorylation of Crk by Abl at Y221 led to the dissociation of Crk from p130Cas. These findings suggest that Abl plays a role in preventing abnormal cell movement and promoting cell death through the phosphorylation of Crk at Y221 and the regulation of the p130Cas-Crk complex. Furthermore, the disruption of this pathway contributed to cell invasion [62].

Overall, p130Cas plays a crucial role in various cellular functions, including survival, proliferation, and migration. Consequently, it is of great importance in pathological processes, such as tumorigenesis and invasion [50]. Collectively, cell invasion and metastasis may be facilitated by the cooperative actions of p130Cas, Crk, and CrkL. The activation of the p130Cas-Crk/CrkL complex-dependent signaling pathways may play significant roles in augmenting invasion and metastasis.

## 6. Conclusion I: Critical Roles of the p130Cas-Crk/CrkL Axis 

**Figure 3:**
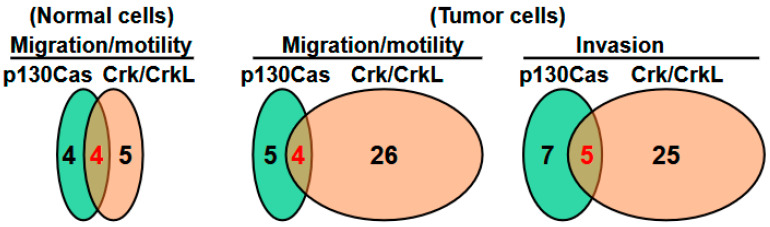
The numbers of the references or citations in which p130Cas and Crk/CrkL were studied separately or combined for cellular functions in normal or tumor cells are indicated as numbers. [Drawn using Microsoft PowerPoint].

p130Cas has been linked to the onset and advancement of numerous types of cancer in humans. Its ability to modulate a wide range of signaling pathways allows p130Cas to participate in various biological processes and transmit signals from growth factor receptor tyrosine kinases, non-receptor tyrosine kinases, and integrins. Moreover, the decrease in tyrosine phosphorylation of p130Cas was observed when cells lost Crk and CrkL. This correlation indicates a strong association between the tyrosine phosphorylation of p130Cas and its binding to Crk and CrkL. The formation of the p130Cas-Crk/CrkL complex is a central event in this intricate process, facilitating downstream signaling cascades that govern cytoskeletal dynamics, ECM remodeling, and cell migration invasion. Dysregulation of this complex has been implicated in the pathogenesis of various cancers, rendering it a promising therapeutic target. This review underscores the critical role of the p130Cas-Crk/CrkL axis in orchestrating cell transformation, migration, and invasion, the hallmarks of cancer. While significant progress has been made, a comprehensive understanding of the molecular mechanisms underlying the p130Cas-Crk/CrkL axis remains elusive. As shown in Table 1 and Table 2, and Figure 3, migration and invasion are the two cellular functions that have been studied the most for the p130Cas-Crk/CrkL axis. Figure 3 indicates that a majority of studies for tumor cell migration and invasion focused on Crk/CrkL only. It is unclear whether Crk/CrkL-dependent tumor cell migration and invasion also depend on p130Cas. It would be interesting to tease out the tumor cell migration and invasion that require both p130Cas and Crk/CrkL from the tumor cell migration and invasion that depend on either p130Cas or Crk/CrkL, but not both. Future studies should delve deeper into the spatiotemporal dynamics of p130Cas, Crk, and CrkL, their interactions with other signaling molecules, and the impact of post-translational modifications. By unraveling these complex processes, we can develop more effective strategies to inhibit cancer cell dissemination and improve patient outcomes.

## 7. Conclusion II: The p130Cas-Crk/CrkL Axis as a Potential Therapeutic Target for Invasive Cancers

This review highlights the importance of developing the inhibitors of the p130Cas-Crk/CrkL axis. So far, most studies have employed genetic manipulations to change the expression levels of p130Cas, Crk, and CrkL proteins. While this approach is valuable in proving the biological roles of individual proteins, it does not address the potential requirement of the collaboration between p130Cas and Crk/CrkL. In other words, it is challenging to distinguish between p130Cas-dependent Crk/CrkL functions and p130Cas-independent Crk/CrkL functions. No chemical or other inhibitors of Crk, CrkL, or p130Cas are currently available, and no concrete approaches to develop such inhibitors have been proposed. Inhibitors of the p130Cas-Crk/CrkL axis, once developed, would enable researchers to tease out the tumor cell migration and invasion that require both p130Cas and Crk/CrkL from the tumor cell migration and invasion that depend on either p130Cas or Crk/CrkL. Inhibitors that specifically block the interaction between p130Cas and Crk/CrkL without affecting the protein levels will enable to establish the contribution of the p130Cas-Crk/CrkL axis to cancer. Such inhibitors would be helpful tools in validating the p130Cas-Crk/CrkL as a potential therapeutic target for invasive cancers in which Crk, CrkL, and p130Cas play critical roles in tumor cell migration and invasion. Developing drugs that inhibit tumor spreading and metastasis can be another layer of therapy to the standard of care that focuses on killing tumor cells or slowing down tumor growth and can improve treatment outcomes for invasive cancers.

## Figures and Tables

**Table 1:** Cellular functions of p130Cas and Crk/CrkL in normal cells.

Normal Cells		
p130Cas Only	Crk/CrkL Only	p130Cas-Crk/CrkL
Cell Morphology [51] Cytoskeleton [3,45,52] Cell Adhesion [51] Cell Migration [8,40,51,53] Cell Invasion [52]	Cell Morphology [54] Cytoskeleton [31,55] Cell Adhesion [56,57] Cell Proliferation [58] Apoptosis [58,59] Cell Differentiation [58,60,61] Cell Motility and Migration [31,56,59,62,63]	Cell Spreading [64] Cytoskeleton [8,65,66] Apoptosis [62] Cell Cycle [67] Cell Migration [8,21,62,64]

**Table 2:** Cellular functions of p130Cas and Crk/CrkL in tumor cells.

Tumor Cells			
p130Cas Only	Crk/CrkL Only		p130Cas-Crk/CrkL
**Cell Spreading:** Breast cancer [76] **Cytoskeleton:** Breast cancer [76,77] **Cell Proliferation:** Breast cancer [70,77,78] Lung cancer [79,80] **Cell Survival and Apoptosis:** Breast cancer [70,77,81] Lung cancer [80] Ovarian cancer [82] **Cell Cycle:** Breast cancer [81] **Cell Transformation:** Lung cancer [79,80] Fibroblasts [52] **Cell Migration:** Breast cancer [76,77] Lung cancer [80] Pancreatic cancer [83] Ovarian cancer [82] **Cell Invasion:** Breast cancer [77,78,84] Lung cancer [80] Pancreatic cancer [85] Ovarian cancer [82]	**Cell Morphology:** Glioma/glioblastoma [86,87] Lung cancer [88] Ovarian cancer [89] Colorectal cancer [16] **Cytoskeleton:** Breast cancer [90] Ovarian cancer [91] Synovial sarcoma [92] **Cell Adhesion:** Glioma/glioblastoma [57,87,93] Breast cancer [57,90,94] Head and neck cancer [95] Pancreatic cancer [96] **Cell Proliferation:** Cervical cancer [97,98,99] Breast cancer [100,101] Glioma/glioblastoma [87,93] Liver cancer [102,103] Lung cancer [104] Synovial sarcoma [105] Rhabdomyosarcoma [106] Gastric cancer [107] Endometrial carcinoma [108] Bladder cancer [109] Head and neck cancer [95] Colorectal cancer [16] **Cell Survival and Apoptosis:** Endometrial carcinoma [108] Cervical cancer [98] **Cell Cycle:** Breast cancer [100] Lung cancer [104] Synovial sarcoma [105] Gastric cancer [107] Endometrial carcinoma [108] **Cell Transformation and Colony Formation:** Liver cancer [102,103,110] Ovarian cancer [89,91] Synovial sarcoma [105] Lung cancer [104] Glioma/glioblastoma [93] Cervical cancer [98] Colorectal cancer [111] Prostate cancer [112] Pancreatic cancer [96] Endometrial carcinoma [108] Breast cancer [57] Fibroblasts [113,114]	**Cell Motility and Migration:** Glioma/glioblastoma [57,86,87,93,115] Bladder cancer [109,116] Ovarian cancer [89,117] Liver cancer [102,110] Colorectal cancer [16,111] Breast cancer [57,90,94] Lung cancer [88,90] Cervical cancer [90,98] Oral squamous cell carcinoma [118] Head and neck cancer [95] Synovial sarcoma [92] Prostate cancer [112] Gastric cancer [119] Pancreatic cancer [96] **Cell Invasion:** Glioma/glioblastoma [57,86,87,115] Ovarian cancer [89,91,117] Lung cancer [88,90,120] Cervical cancer [90,97,98] Breast cancer [57,90,94] Gastric cancer [119,121] Liver cancer [102,110] Colorectal cancer [16,111] Bladder cancer [109] Prostate cancer [112] Oral squamous cell carcinoma [118] **Epithelial-Mesenchymal Transition:** Breast cancer [101] Bladder cancer [109] Colorectal cancer [111] Gastric cancer [119] Lung cancer [122] Ovarian cancer [117]	**Cell Transformation:** Glioma/glioblastoma [25] Fibroblasts [123] **Cell Migration:** Prostate cancer [124] Glioma/glioblastoma [25] Breast cancer [125] Fibrosarcoma [18] **Cell Invasion:** Breast cancer [125] Glioma/glioblastoma [25] Pancreatic cancer [62] Prostate cancer [124] Colorectal cancer [47]
